# Retrons and their applications in genome engineering

**DOI:** 10.1093/nar/gkz865

**Published:** 2019-10-10

**Authors:** Anna J Simon, Andrew D Ellington, Ilya J Finkelstein

**Affiliations:** Center for Systems and Synthetic Biology and Department of Molecular Biosciences, University of Texas at Austin, Austin, Texas 78712, USA

## Abstract

Precision genome editing technologies have transformed modern biology. These technologies have arisen from the redirection of natural biological machinery, such as bacteriophage lambda proteins for recombineering and CRISPR nucleases for eliciting site-specific double-strand breaks. Less well-known is a widely distributed class of bacterial retroelements, retrons, that employ specialized reverse transcriptases to produce noncoding intracellular DNAs. Retrons’ natural function and mechanism of genetic transmission have remained enigmatic. However, recent studies have harnessed their ability to produce DNA *in situ* for genome editing and evolution. This review describes retron biology and function in both natural and synthetic contexts. We also highlight areas that require further study to advance retron-based precision genome editing platforms.

## INTRODUCTION

The ability to precisely edit genomic loci has been a long-standing goal of biotechnology. Targeted genome editing is being exploited to probe genotype-phenotype relationships, repair disease-causing alleles, develop designer organisms with modified genomes, and to biologically record intra- and extracellular conditions. Classically, strain improvement has been achieved either via non-specific mutagenesis of genomes (1,2) or via targeted modifications of specific genes expressed on plasmids (3,4). Recombineering (5) and CRISPR-based genome editing (6,7) have dramatically increased the ease and throughput of targeting individual genomic loci. Multiplexing and iterating recombineering- and CRISPR-based editing technologies have enabled high-throughput exploration of genotype–phenotype relationships and continuous evolution of synthetic genomes with novel properties, for example in Multiplex Automated Genome Engineering (‘MAGE’) (8), Yeast Oligo-mediated Genome Engineering (‘YOGE’) (9), and iterative CRISPR EnAbled Trackable genome Engineering (‘iCREATE’) (10).

Retrons are a potentially useful tool for targeted genomic engineering because they produce intracellular DNAs via reverse transcription of noncoding structural RNAs (11,12). While the natural biology of these widespread retroelements is largely unknown, retrons have recently been repurposed as *in situ* sources of donor DNA for both recombineering- and CRISPR-based genome editing applications (13–15). This production of intracellular DNA has so far enabled recording of cellular states (13), high-throughput genetic editing (14), and targeted genome mutation (15). Here, we review retron biology and retron-enabled genome engineering applications. We conclude with a discussion of future prospects and challenges in retron-based genome editing.

## RETRON BIOLOGY

### Retron discovery and biochemistry

**Figure 1.**
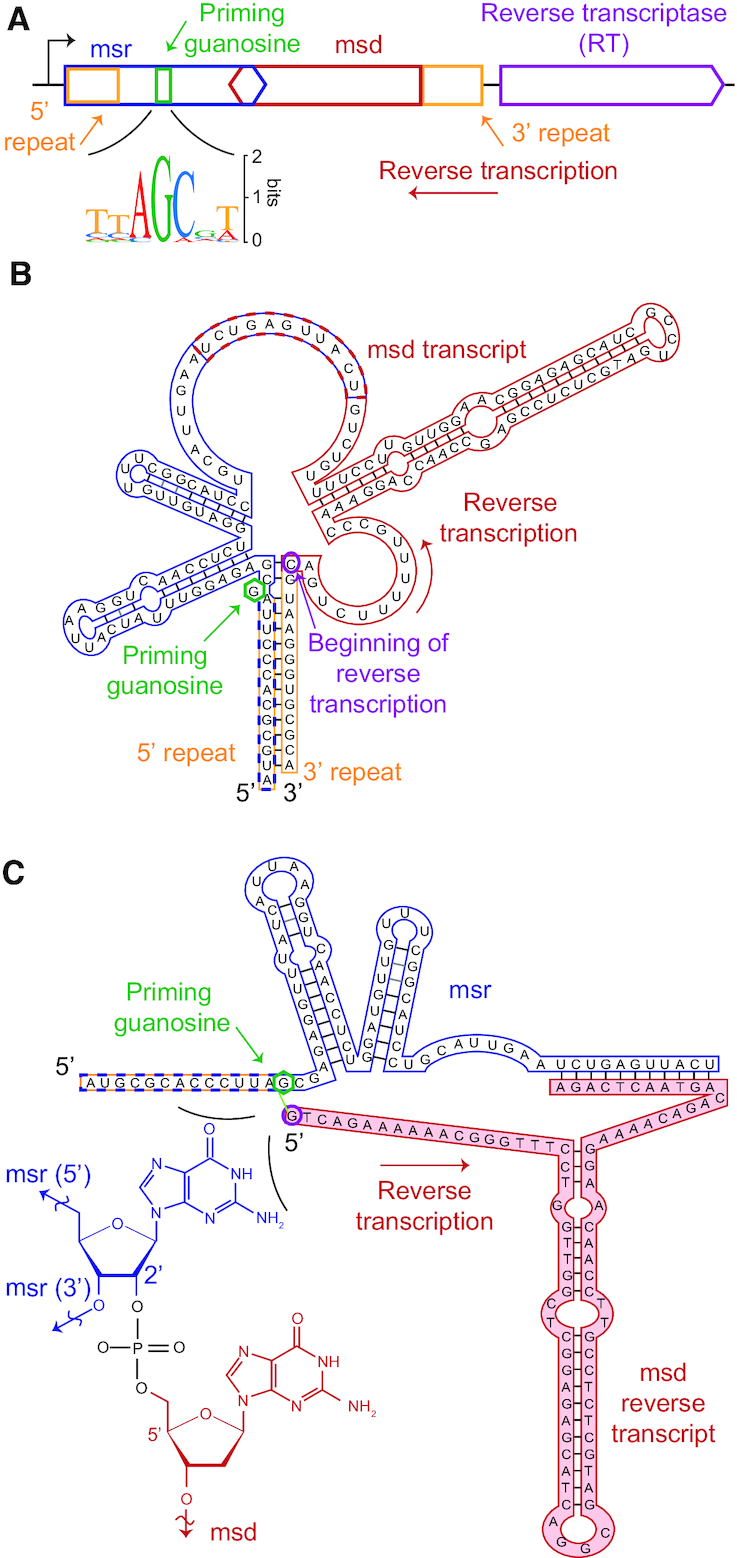
Retron structure and organization. (**A**) Retrons are encoded as a single polycistronic transcriptional cassette containing a promoter, an msr (blue) including a conserved priming guanosine residue (green), an msd (red), self-complementary regions (yellow), and a reverse transcriptase (purple, not to scale) (12). The black arrow shows the direction of the coding strand for the retron cassette; the red arrow shows the direction of reverse transcription. Inset: sequence logo of nucleotides adjacent to the priming guanosine of 15 msr-msd sequences. Retron-Sen1 (Se72) was excluded because its msr lacks an apparent priming guanosine (46). (**B**) Structure of a representative msr-msd transcript encoded by Retron-Eco1 (Ec86) (23). The transcript encoding the reverse transcriptase is cleaved from that of the msr-msd before folding (12). The cytosine at which reverse transcription begins is circled in purple. (**C**) Structure of the mature Retron-Eco1 (Ec86) msDNA. Inset: the msr and the 5′ end of the reverse-transcribed msd are covalently bonded to the priming guanosine via a 2′-5′ linkage.

Retrons were initially discovered in 1984 from the presence of short satellite RNA–DNA molecules in bacterial DNA preparations (16). Gel electrophoresis of phenol-extracted chromosomal DNA from *Myxobacteria xanthus* and *Stigmatella aurantica* indicated a secondary satellite band with a mobility of ∼120–190 base pairs (bp) (16). Subsequent studies revealed that these bands, termed multicopy single-stranded DNAs (msDNAs), are comprised of one strand of structured RNA, the ‘msr,’ connected to one strand of DNA, the ‘msd.’ The msr and msd molecules are joined by a 2′-5′ phosphodiester bond between a priming guanosine within a conserved AGC sequence in the msr and the phosphate of the 5′ end of the msd that covalently links the RNA and DNA strands into a single branched molecule (Figure 1). The msr and msd are encoded in a compact, contiguous transcriptional cassette that also includes a specialized reverse transcriptase (RT); we refer to this cassette as a whole as the retron. The discovery of retron RTs was particularly notable because they were the first discovered bacterially-encoded RTs (17). *In vitro* studies demonstrated that *Escherichia coli* retron RTs and their paired msrs were both necessary and sufficient to produce msDNA with the characteristic 2′-5′ linkage (18,19). To date, 38 distinct retrons have been identified experimentally by their production of msDNA; 16 of these, which we subsequently refer to as the ‘experimentally validated retrons’, contain fully annotated and experimentally-validated msr-msd-RT cassettes. Table 1 summarizes all experimentally-characterized retrons reported to date, along with their host organisms. Bioinformatic approaches have also identified hundreds of putative retrons that have not been characterized experimentally (20–22).

**Table 1.** Summary of all retrons whose production of msDNA has been experimentally validated. The new systematic nomenclature is described in the main text. In parenthesis, we also include the names initially developed in (23). The 16 shaded entries, referred to as the ‘experimentally validated retrons’, contain fully annotated and experimentally-validated msr-msd-RT cassettes listed in the National Center for Biotechnology Information (NCBI) databank (accessed August 2019). The 22 remaining entries contain elements that have been experimentally demonstrated to produce msDNA but do not correspond to fully annotated or validated entries in the NCBI databank

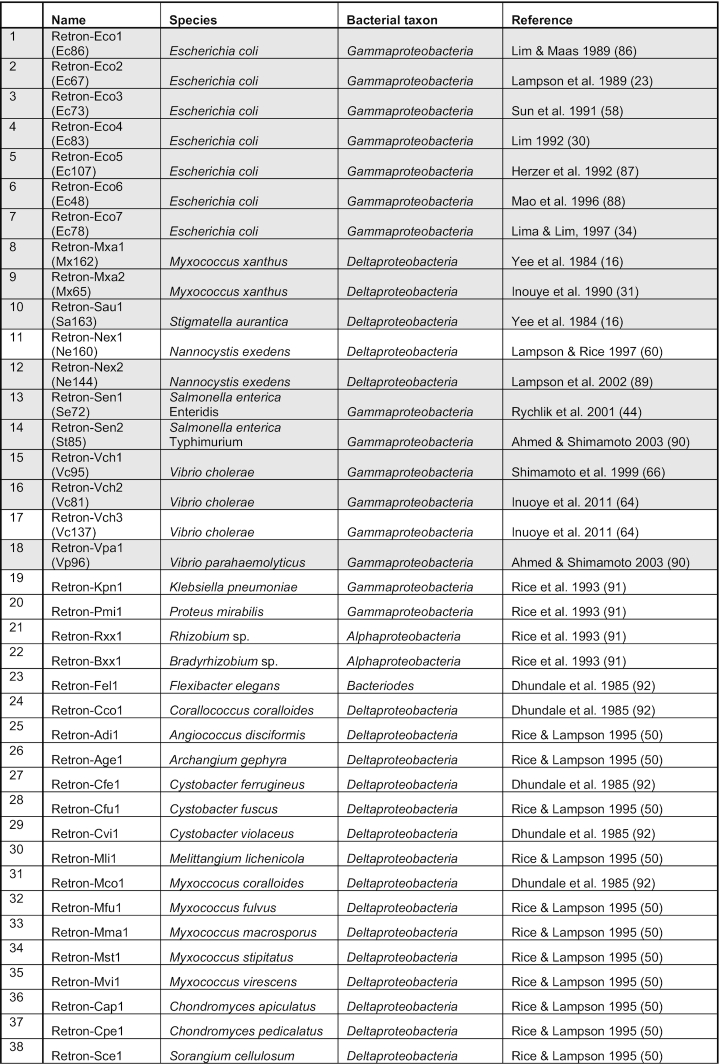

The rapid discovery of hundreds of putative retrons has prompted us to develop a systematic naming convention for these retroelements. Currently, retrons are named by the first letters of their genus name, species name, and the length of reverse-transcribed DNA in their native msDNA sequence. This convention was initially developed by Inouye *et al.* in 1989, who termed a retron isolated from *E. coli* that contained 86 bases of reverse-transcribed DNA ‘Ec86’ (23). However, this nomenclature is not easy to adapt for large-scale, systematic retron discovery for three reasons: (i) the length of the msd of most experimentally validated and putative retrons is unknown; (ii) two letters are insufficient to uniquely identify the genus and species of many retron-encoding bacteria; and (iii) the convention does not distinguish the entire retron from the RT. Therefore, we propose a more general naming convention that is adapted from the restriction enzyme literature (Table 1) (24).

- Retrons are named as the first letter of their genus, first two letters of their species, and an Arabic numeral corresponding to the order with which they were discovered relative to other retrons from the same species or a different species with the same first genus and first two species letters, respectively.
- The prefix ‘Retron-’ is added to signify that this object is a retron, and the prefixes ‘RT-’, ‘msr’, ‘msd’ and ‘msDNA’ are added when referring to the reverse transcriptase enzyme, msr, msd and msDNA, respectively.

Under the new convention, ‘Ec86’ is re-named ‘Retron-Eco1’ and the corresponding RT is ‘RT-Eco1.’ For clarity and continuity, the rest of this review will use the new convention and will also include the historical names in parenthesis (e.g. Retron-Eco1 (Ec86)).

### Msr, msd and msDNA structure and production

In all experimentally validated retrons, the msr and msd are encoded within a single transcriptional cassette, with the msr to the 5′ of the msd (Figure 1A). The retron msr-msd transcriptional cassettes are divergent at the sequence level but share substantial structural homology (Supplementary Figure S1). The structural elements are likely necessary for function. Prior to reverse transcription, the msr and msd form a single highly structured transcript (Figure 1B). The msr structure contains 1–3 stable hairpins with ∼7–10 bp-long stems and ∼3–10 nt loops (12,25). The msd-encoding segment of the transcript is also highly structured, folding into a single hairpin with a long (∼10–50 bp) stem that generally contains mismatched or wobble base pairs. In addition to this internal structure of the msr and msd, the ∼8–30 bases at the 5′ end of the msr and the 3′ end of the msd transcript are complementary and hybridize (Figure 1B). The internal (e.g. 3′) and of the msr portion of this stem contains or ends immediately 5′ to the conserved guanosine residue that primes reverse transcription of the satellite DNA. Most commonly, this guanosine is found within a conserved TAGC sequence, though occasionally this sequence is CAGA (as in Retron-Eco2 (Ec67)), CAGC (as in Retron-Sau1 (Sa163) and Retron-Mxa1 (Mx162)), GAGC (as in Retron-Mxa2 (Mx65)) or ACGC (as in Retron-Vch2 (Vc81)) (Figure 1A, inset).

The RT initiates reverse transcription using the base-paired 5′ and 3′ stem of the msr-msd as a primer (12). Early experiments demonstrated that several *E. coli* retron RTs and their paired msrs are both necessary and sufficient to produce msDNA with the characteristic 2′-5′ linkage (18,19), and that the 2′-5′ linkage between the priming guanosine in the msr and first base of the msd DNA is catalyzed directly by the RT (26). The RT extends the reverse transcript until a defined position at which reverse transcription stops *in vitro* and *in vivo* via an unknown mechanism. The observation that the RT terminates at similar sites *in vitro* and in cells strongly suggests that RNA structure directs RT termination. Concurrently with reverse transcription, cellular RNAse H activity degrades the template of the msd, excluding ∼5–10 RNA bases at its 5′ end. This segment of the RNA remains hybridized to the complementary reverse transcript and is thus be considered to be part of both the msr and msd (27) (Figure 1C). The mechanism limiting RNAse H activity at terminal msd residues also remains unexplored.

Retron msDNA is further nucleolytically processed after reverse transcription (12,17,28,29). Often, some or all of the msr upstream of the conserved priming guanosine is degraded. For example, the Retron-Mxa1 (Mx162) msDNA is initially transcribed as a 241 nucleotide (nt) transcript. In the mature msDNA, 16 residues are cleaved from the 5′ end, leaving a 71-nt msr and a 162-nt msd msDNA (the msr and msd overlap at 8 bases) (17). Other retrons undergo much more dramatic processing that results in a mature msDNA that is primarily reverse transcribed DNA. For example, msDNA-Eco4 (Ec83) is endonucleolytically cleaved between the fourth and fifth nucleotides from the priming guanosine residue, removing that linkage to the msr (12,28). This cleavage likely results in the dissociation of the base pairs at the 3′ ends of the msd and msr, since 90% of experimentally-observed msDNA-Eco4 (Ec83) is not joined to RNA (30). While the enzyme responsible for this cleavage is not known, it may arise from a putative ATP-binding protein encoded just downstream of RT-Eco4 (Ec83) in the native cassette.

Reverse transcription requires both an msr-msd sequence and its cognate RT. Expressing the RT of one retron and the msr of another does not produce msDNA (31–33)), with some exceptions for highly homologous retrons (34,35). Mutational and Systematic Evolution of Ligands by Exponential Enrichment (SELEX) -based studies of Retron-Eco1 (Ec86) and Retron-Eco3 (Ec73) indicated that both msr sequence and secondary structure are important for RT recognition. While RT-Eco1 (Ec86) can bind moderately truncated or modified variants of its cognate msr, removing or destabilizing a core section containing an 8 bp stem and a three base loop abrogates binding. Similarly, RT-Eco3 (Ec73) binds a specific 9 bp stem, three base loop section of its cognate msr but fails to bind other msr truncations (32,36).

### Reverse transcriptase structure and function

**Figure 2.**
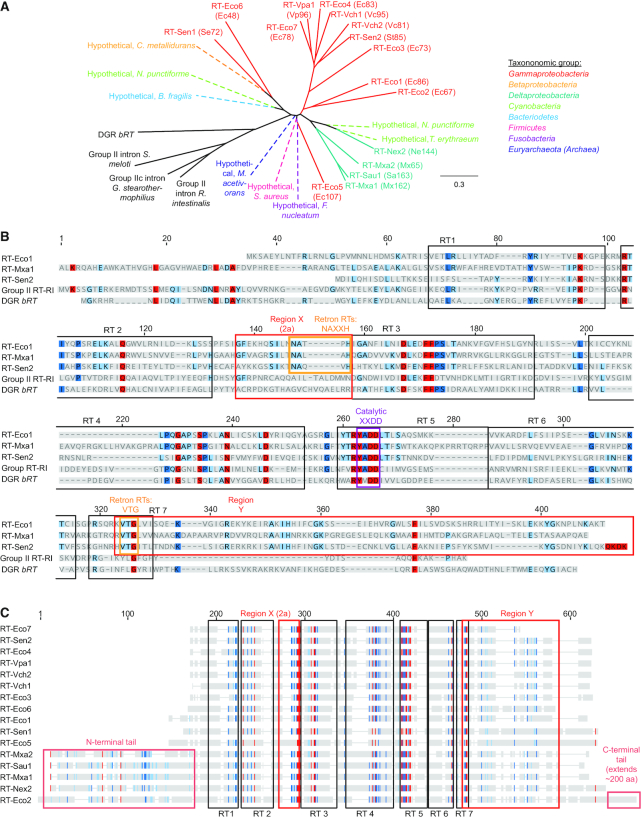
Retrons form a distinct class within the larger group of bacterial RTs. (**A**) Phylogenetic tree showing relationships between Group II intron maturase reverse transcriptases, *bRT* RT from the *Bordetella* bacteriophage Diversity-Generating Retroelement (DGR), and retron RTs. Group II intron maturase and DGR RTs are colored black; retron RTs are colored by phylum. Putative retrons (dashed) identified from *Bacteroidetes*, *Betaproteobacteria*, *Cyanobacteria*, *Fusobacteria*, *Firmicutes*, and *Euryarchaeota* genomes were employed as these groups lack experimentally validated retrons (39). (**B**) Sequence alignments of RT-Eco1 (Ec86), RT-Mxa1 (Mx162), RT-Sen2 (Se72), *Roseburia intestinalis* Group II intron maturase reverse transcriptase domain (‘Group II RT-RI’), and the *Bordetella* bacteriophage DGR reverse transcriptase *bRT*. Each retron RT sequence aligns well to the seven universal reverse transcriptase regions (boxed in black) and includes several interdomain regions, including the retron-specific regions X and Y (boxed in red) (12,40). Conserved, retron-specific residues in regions X and Y are boxed in orange; the catalytic XXDD core common to all known reverse transcriptases is boxed in purple. (**C**) Distant-level view of the alignment of RT sequences from all 16 fully characterized retrons. Sequences were aligned with the NCBI online tool COBALT (38); the alignment was then employed to construct a phylogenetic tree using the neighbor-joining tree build method in Geneious software under a Jukes-Cantor genetic distance model. Residues shaded in red, dark blue, light blue, and grey correspond to residues that are 100%, 80–100%, 60–80% and <60% homologous across the aligned sequences. RT-Mxa1 (Mx162), RT-Mxa2 (Mx65), RT-Sau1 (Sa163), and RT-Nex2 (Ne144) contain an extended N-terminus; RT-Eco2 (Ec67) contains both N- and C-terminal extensions (boxed in light red).

Retron RTs form a diverse class within the broader family of bacterial RTs (Figure 2, Supplementary Figure S2). Early estimates suggested that retrons encode 11–14% of all bacterial RTs, second only to Group II intron maturases (20,21). More recently, a metagenomic survey suggested that diversity-generating retroelements (DGRs) may be more common than retrons in uncultivated bacteria (37). Nonetheless, retron RTs are quite common in diverse bacteria (see next section). Experimentally validated retron RTs share just ∼14% average amino acids (aa) homology with the *Roseburia intestinalis* Group II intron maturase RT domain, ∼12% with the *Bordetella* bacteriophage DGR *bRT* RT, and *∼*25% homology with each other (Supplementary Table S1).

Phylogenetic analysis indicates several distinct groups of retron RT sequences, which together form a group distinct from RTs associated with both Group II intron maturases and DGRs (Figure 2A). To identify the relationship between retron RTs and other bacterial RTs we first aligned the sequences of all 16 fully annotated retron RTs, eight previously putatively identified retron RTs from taxonomic groups lacking experimentally validated retrons, three Group II intron maturase RTs, and the *Bordetella* bacteriophage DGR *bRT* using NCBI’s Constraint-based Multiple Alignment Tool (COBALT) (38). We then built a phylogenetic tree from these alignments using the Geneious Tree Builder tool with the Jukes-Cantor genetic distance model and the neighbor-joining tree build method. In the resulting tree, retron RTs branch from the Group II intron maturase and DGR RTs. Notably, the putative retron RT sequences, which originated from genomes of bacteria in the *Bacteroidetes*, *Betaproteobacteria*, *Cyanobacteria*, *Fusobacteria*, and *Firmicutes*, and the archaeon *Methanosarcina acetivorans* (39), and the experimentally validated retron RT sequences, which originated only from *Gammaproteobacteria* and *Deltaproteobacteria*, are clearly within this group separate from other bacterial RTs. There are several clear groups within the retron RTs. One group contains the myxococcal RT-Mxa2 (Mx65), RT-Mxa1 (Mx162), RT-Nex2 (Ne144) and RT-Sau1 (Sa163) and putative cyanobacterial retron RTs from *Nostoc punctiforme* and *Trichodesmium erythraeum*. Another group contains RT-Vch1 (Vc95), RT-Eco4 (Ec83), RT-Vpa3 (Vp96), and RT-Eco7 (Ec78). The remaining RTs in this analysis do not fall into closely related groups.

Examination of the aligned retron RT sequences highlights the seven conserved RT motifs and two retron-specific inter-motif regions (Figure 2B, Supplementary Figure S2) (40). Most retron RTs are ∼300–400 aa long and lack bioinformatically identifiable endonuclease, maturase, or RNAse domains (Figure 2C) (12,23). Several retron RTs also encode additional N- and C-terminal extensions of unknown function. For example, myxococcal retron RTs (RT-Mxa1 (Mx162), RT-Mxa2 (Mx65), RT-Sau1 (Sa163) and RT-Nex2 (Ne144)) contain an extended N-terminal region. Similarly, RT-Eco2 (Ec67) is 586 residues long and contains both N- and C- terminal extensions that lack homology to non-retron protein domains.

The conserved RT motifs include the ‘palm’ and ‘fingers’ domains and the conserved double aspartic acid-containing catalytic core. Retron RTs encode the YADD catalytic tetrad, as seen in Group II intron maturase RTs and other bacterial RTs. As with other aspects of the retron RT sequence, several variants encode altered catalytic cores (e.g., YCDD in RT-Eco5 (Ec107), Supplementary Figure S2). Of the two conserved retron-specific motifs, region X is a ∼16 aa segment that is between RT motifs 2 and 3 (21,22). Fourteen of the 16 experimentally-validated retron RTs contain an NAXXH aa sequence in region X. The two outliers, RT-Eco6 (Ec48) and RT-Mxa2 (Mx65)), contain substitutions at the asparagine residue (Supplementary Figure S2). Notably, while both Group II intron and DGR RTs likewise contain residues between motifs 2 and 3, they lack the characteristic NAXXH motif seen in retron RTs. Deletion of region X from RT-Eco1 (Ec86) and RT-Eco3 (Ec73) does not block RT-cognate msr binding, but abrogates synthesis of the msDNA product, suggesting that it may be essential in reverse transcription initiation or catalysis (32). Notably, structures of Group II intron maturase RTs indicate that the equivalent region 2a forms an alpha helix that may stabilize the β hairpin containing the active site, influencing catalysis (41,42). We conjecture that region X in retron RTs may likewise stabilize the active site to promote catalysis.

Region Y is a ∼90 aa segment at the C-terminus of retron RTs. Region Y is defined as beginning within motif 7 at a highly conserved VTG triplet and extending to the C-terminal end of the protein (12,32). Fourteen experimentally verified retron RTs contain the VTG triplet; RT-Vch2 (Vc81) and RT-Eco6 (Ec48) contain a V→I and T→H substitutions, respectively. Beyond the conserved VTG, region Y is variable. Biochemical studies indicate that region Y directs the RT to its cognate msr. Deleting region Y from RT-Eco1 (Ec86) and RT-Eco3 (Ec73) abrogated their ability to bind their msrs (32). Moreover, a purified C-terminal fragment of region Y of RT-Eco1 (Ec86) (residues 255–320) bound the stem-loop of msr-Eco1 (Ec86) with a *K_D_* of ∼5 × 10^−8^ M, as well as a shorter fragment containing an eight base-pair stem of the msr-Eco1 (Ec86) with a three base UUU loop, but not the msr-Eco3 (Ec73). Likewise, a purified C-terminal fragment of RT-Eco3 (Ec73) containing its region Y (residues 251–311) bound the cognate msr-Eco3 (Ec73) msr tightly (though the specific *K*_D_ was not measured), but not msr-Eco1 (Ec86) msr (36). Swapping region Ys between RT-Eco1 (Ec86) and RT-Eco3 (Ec73) produced chimeric proteins with ‘swapped’ msr recognition (32). However, all region Y studies to date have focused on Retron-Eco1 (Ec86) and Retron-Eco3 (Ec73). Additional biochemical experiments will be required to confirm that region Y broadly recognizes the stem of its cognate msr in diverse retrons.

### Accessory open reading frames in retron cassettes

**Figure 3.**
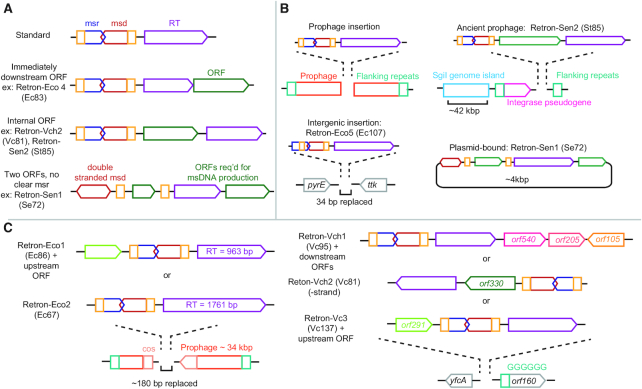
Organization and genomic context of select retron cassettes. (**A**) Structures of representative validated retron cassette genes. The msr (blue), msd (red), complementary repeat regions (yellow), and RT (purple), and associated unknown ORFs (green) are indicated. Many retron cassettes (‘standard’) consist only of an msr and msd flanked by complementary regions upstream of an RT. Variations on this theme include ORFs either immediately downstream of the RT or between the msr-msd and the RT. Retron-Sen1 (Se72) contains an msd that is transcribed in both directions to produce double-stranded DNA. A hairpin-containing ORF flanked by repeat regions may function as an alternative msr. The cassette also includes an RT and a second unknown ORF with homology to cold shock proteins (46). (**B**) Genomic contexts of retron cassettes. Several validated retrons occur in putative prophage sites flanked by repeat regions (12). Retron-Sen2 (St85) occurs in what is thought to be an ancient prophage site downstream of the Sgi1 genomic island associated with pathogenesis. Retron-Eco5 (Ec107) replaces 34 bases of an intergenetic region with no apparent mobile element nearby. Retron-Sen1 (Se72) occurs on a plasmid. (**C**) Several different retrons, along with additional orfs, may occupy the same locus in different bacterial strains or populations. For example, the Retron Eco1-(Ec86) and an upstream ORF occupies the same genetic site as Retron-Eco2 (Ec67) in different *E. coli* strains (59,85). The *Vibrio cholerae* retrons Retron-Vch1 (Vc95), Retron-Vch2 (Vc81) and Retron-Vch3 (Vc137) also occupy the same genetic site.

Approximately a third of annotated retrons encode accessory open reading frames (ORFs) of unknown function (Figure 3A). These ORFs may be between the msr-msd and the RT (e.g., in Retron-Eco3 (Ec73), Retron-Sen2 (St85), and Retron-Vch2 (Vc81)) or immediately 3′ of the RT (e.g. in Retron-Eco4 (Ec83)). Some of these ORFs are putatively annotated as adenosine-binding proteins, (e.g., in Retron-Eco4 (Ec83) (43)) or cold-shock proteins (e.g. in the *S. enterica* Enteridis Retron-Sen1 (Se72)) (44). Notably, some of the retrons with accessory ORFs also produce unusual msDNAs. For example, Retron-Eco4 (Ec83) produces single-stranded DNA via site-specific cleavage of its msDNA (45). Retron-Sen1 (Se72) shows an even more dramatic departure from the retron paradigm; the 2′-5′ linkage is not experimentally observed (44,46). Here, the RNA template is degraded after the first round of RT, and the single-stranded DNA is used as a template to produce double-stranded DNA (dsDNA). RT-Sen1 uses the free 3′OH end of the msr to initiate the second round of DNA synthesis. Despite the unusual dsDNA product, RT-Sen1 (Se72) is structurally homologous with other retron RT sequences. Although it is tempting to speculate that the presence of accessory ORFs is important for producing unusual msDNA variants, additional biochemical studies are required to define their functions. These enigmatic ORFs may also provide opportunities for basic discovery and biotechnological applications.

### Biological distribution and transmission

Experimental and bioinformatic studies indicate that retrons are sporadically distributed throughout the bacterial kingdom. Experimentally, msDNA has been isolated from a diverse subset of *Alpha-*, *Delta-* and *Gammaproteobacteria* (Table 1) (47). Screens of msDNA production, however, have commonly indicated that msDNA is not consistently produced by all strains within these taxonomic groups. Screens of *E. coli* identified msDNA in four of 89 and seven of 113 clinical and environmental isolates (48,49). Another study identified msDNA from 4 of 23 *Proteus mirabilis* isolates; 1 of 21 *Klebsiella pneumoniae* isolates, and 10 of 63 *Rhizobia* isolates (49). In contrast, msDNA production appears very common within the myxobacteria, with msDNA found in 27 of 28 tested myxobacterial strains (50).

In addition to these experimentally-identified retrons, bioinformatic surveys have identified putative retrons in *Cyanobacteria*, *Fusobacteria*, *Bacteroidetes, Firmicutes* (39), *Actinobacteria* (21,51) and even in the archaeon *Methanosarcina acetivorans* (39). Typically, these studies first identify retron-specific RTs in gene or protein databanks (20,21), metagenomic data (52,53), or in newly-sequenced bacterial genomes (51,54). Recent studies have additionally examined the regions upstream of the RT for palindromic and hairpin-containing motifs similar to msr-msd regions of experimentally validated retrons (55,56).

The sporadic distribution of retrons within prokaryotic taxonomic groups and the mismatch in GC% content from their host strains suggest lateral transmission (12). While retrons do not appear to encode elements that support genetic mobility (e.g., RT endonuclease domains such as those required for the mobility of Group II introns), many are associated with mobile elements and prophage insertion sites (Figure 3B) (12). For example, Retron-Eco3 (Ec73) is inserted into a bacteriophage-like element termed φR73 integrated into the chromosomal selenocystyl-tRNA locus. Infection of φR73-containing *E. coli* cells with P2 phage produced infectious virions that lysogenized new host strains, resulting in the integration of φR73 at the selenocystyl-tRNA locus and production of msDNA-Eco3 (Ec73) (57,58). Retron-Eco1 (Ec86) and Retron-Eco2 (Ec67) both replace an identical ∼180 bp between a phage cohesive end site and the terminator of a DNA packaging gene (59) (Figure 3A). Retron-Sen1 (Se72) even exists on a bacterial plasmid (44). Retron-Nex1 (Ne160) contains several apparently duplicated repeats of msr and msd sequences without an RT within a kilobase, suggesting that this element may have been repeatedly duplicated and inserted by a distant RT or alternative element (60). Other retrons, however, are not associated with obvious prophage or other mobile elements. Retron-Eco5 (Ec107), for example, replaces a palindromic 34 bp in an intergenic region that lacks any further characteristics of mobility or prophage insertion. In addition to horizontal transfer, many myxococcal retrons, including Retron-Mxa1 (Mx162) and Retron-Sau1 (Sa163), cluster in closely related bacterial species and show a codon bias that is similar to that of the host. These retrons likely arose or were acquired in a common ancestor of the myxobacteria and were vertically inherited (50).

### Hypothesized biological functions

Retrons’ functional role—if any—remains poorly understood. Some retrons could potentially be purely selfish genetic elements that are neutral or weakly deleterious to the host. However, the observation that retrons encode functional proteins suggests that they may be beneficial to the host, possibly under specialized conditions (61). In the following paragraphs, we discuss the retrons with known functions and consider potential roles for those whose function remains unknown.

Circumstantial evidence suggests that some retrons may play key roles in pathogenesis. Disabling Retron-Sen2 (St85) or Retron-Eco4 (Ec83) by erasing their ability to produce msDNA reduced the ability of their enteropathogenic *S. enterica* Typhimurium and *E. coli* hosts to persistently colonize mouse intestine (62). Closer studies of the msDNA-null *S. enterica* Typhimurium cells indicated severe growth defects in anaerobic conditions. Growth was rescued by the addition of nitrate, a terminal electron acceptor typically present in aerobic environments. Proteome analysis indicated differential expression of 238 proteins between wild-type and msDNA-lacking *S. enterica* Typhimurium under anaerobic conditions, most involved in the reduction of electron acceptors present in anaerobic environments. While the patterns of protein regulation did not follow those associated with the major known regulatory proteins in *S. enterica* Typhimurium, the authors concluded that in some genetic or epigenetic way Retron-Sen2 (St85) controls protein abundance that in turn enables the use of the electron acceptors present in anaerobic environments. Conversely, other studies found that deletion of RT-Sen2 (Se85) from other, multidrug-resistant strains of *S. enterica* Typhimurium increased virulence, suggesting an opposing role in the regulation of virulence for this retron (63).

The apparent phylogenetic subgroup obtained from aligning RTs that includes Retron-Sen2 (St85) and Retron-Eco4 (Ec83) also contains retrons from other pathogenic bacterial serovars, including Retron-Eco7 (Ec78), Retron-Vch1 (Vc95), and Retron-Vpa3 (Vp96). The RTs associated with these retrons have relatively similar sequences, with aa identity of ∼40–58% (Supplementary Table S1). Several also contain accessory ORFs between the msd and RT or downstream of the RT (62,64–66) (Figure 3A). Interestingly, the msrs of two retron pairs in this group, msr-Vch1 (Vc95)/msr-Vpa1 (Vp96) and msr-Eco4 (Ec83)/msr-Eco7 (Ec78), share ∼75% and ∼60% sequence identity (63). Correspondingly, RT-Vch1 (Vc95) and RT-Eco4 (Ec83) recognize and reverse transcribe msr-msd-Vpa3 (Vp96) and msr-msd-Eco7 (Ec78), respectively (35). These are the only two examples where an RT can function on a non-cognate msr-msd. Moreover, Retron-Vch1 (Vc95) is almost exclusively found in the epidemic cholera-causing serotypes (O1 and its derivative O139); this retron is very rare in non-pathogenic serotypes (66). In contrast, two non-pathogenic serotypes contain alternate retrons in the same genomic locus—Retron-Vch2 (Vc81) and Retron-Vch3 (Vc137). Retron-Vch2 (Vc81) is also oriented in the opposite direction from Retron-VchI (Vc95) (64). The presence of certain retron variants only in pathogenic species, and the similarities between such retrons, has led to speculation that retrons may play a mechanistic role in pathogenesis (35). Alternatively, the ORFs of unknown function could themselves be pathogenicity determinants that associate with retrons to hitchhike between genomes (Figure 3C).

Beyond potential roles in pathogenesis, retrons may play adaptive roles in the utilization of non-traditional energy sources. For example, expression of Retron-Eco5 (Ec107) is strongly enhanced in cells that are starved for phosphate or entering stationary phase in rich media (67). Similarly, Retron-Eco1 (Ec86) expression resulted in significant downregulation of eight genes and upregulation of one gene. The eight downregulated genes were important in carbon utilization; the remaining gene upregulated the production of the ribosomal protein S2 (65). Beyond non-pathogenic *E. coli*, retrons are especially common in myxococcal bacteria that undergo significant morphological changes, forming multicellular-like, ‘fruiting’ bodies in response to nutrient insufficiency, and show complex intercommunity behavior and predation (68); perhaps retrons in these species help enable these phenotypic changes. Additional studies are required to link retron expression and cell specialization.

While there are several hypotheses about retrons’ biological roles, there is little known about their mechanism of action. One possibility is that retrons modulate host DNA-binding proteins via binding to the msDNA. Overexpression of some *E. coli* retrons producing DNA with stems containing mismatched base pairs increases genomic mutation rates by ∼40-fold (69) and recombination rates in high-frequency recombination (Hfr) crosses with a competent *S. enterica* Typhimurium donor strain ∼5–10-fold (70). This increase in genomic mutation rate likely occurs because the mismatch repair protein MutS is sequestered onto the mismatch-containing msDNA stem, reducing protein availability for genomic replication-associated mismatch repair. Notably, overexpression of retrons that produce msDNA with fully complementary stems does not increase mutation or Hfr rates (70,71). While these increased mutation frequencies only occurred after ∼100-fold overexpression of the msDNA relative to standard lab growth conditions, this study may mimic intracellular or environmental conditions that increase retron expression to transiently increase genomic mutation rates (69). msDNA may sequester other DNA-binding proteins to regulate their activity and, in turn, gene expression patterns (72,73). Additionally, msDNA may directly act as a post-transcriptional gene regulator, similarly to microRNAs or RNA-binding proteins (74).

Finally, retrons might play a role in targeted genome diversification or adaptive immunity. DGRs are found in bacteria, viruses, and archaea and selectively diversify targeted loci known as ‘variable’ regions (75). These variable regions typically encode surface-displayed peptides that bind a changing set of ligands in their environments (76,77). To target and diversify these regions, DGRs employ specialized RTs to reverse transcribe homologous regions known as ‘template’ regions, incorporating random nucleotides opposite from adenine residues on the RNA transcript intermediate. The mutated reverse transcript then specifically retrotransposes to the variable region. While the details of reverse transcription and transposition have yet to be determined, recent studies have found that, like retron-produced msDNA, the prototypical *Bordetella* bacteriophage *brt* RT produces a branched RNA-DNA structure most likely joined by 2′-5′ phosphodiester bonds (78,79). These similarities between retron msDNA and DGR cDNA suggest that perhaps retron msDNA may home to genetic sequences via a mechanism that is analogous to DGR retrohoming. Alternatively, a recent study suggests that certain CRISPR-related RTs may have arisen from retron-like RTs, suggesting that retrons could potentially play a similar role in adaptive immunity or integration of specific RNA or DNA sequences into defined genomic positions (80).

## RETRONS IN BIOTECHNOLOGY

Retrons are potentially powerful tools for genome editing because they can produce high copy number intracellular DNAs in orthogonal hosts. Early experiments indicated that a Retron-Eco2 (Ec67) msr and RT could successfully reverse transcribe a Retron-Eco3 (Ec73) msd, and vice-versa (33). This critical experiment defined that while a retron's msr and RT are paired, the msd is variable and can encode an intracellular DNA with an arbitrary sequence. This critical insight enabled the repurposing of retrons for biotechnological applications. To date, four studies have employed retrons for gene silencing or genome editing (13–15,81). Below, we discuss the context, central findings, and potential further development of these studies.

### Gene targeting in bacteria

An early study demonstrated that retrons can be engineered to produce antisense cDNAs that hybridized to and ultimately silenced, mRNAs of the targeted genes. Shimada and colleagues replaced a portion of the msd of Retron-Eco5 (Ec107) with a sequence targeting the translation initiation sites of an mRNA encoding the *E. coli* outer membrane lipoprotein (81). The authors tested two different targeting sequences, as well as a targeting sequence containing an EcoRI digestion site, potentially enabling cleavage of the sequence targeting lipoprotein. Each targeting sequence inhibited lipoprotein production ∼75%.

**Figure 4.**
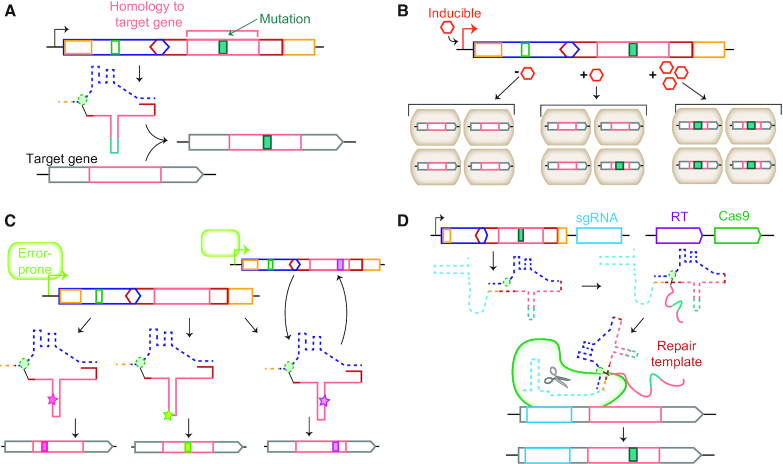
Biotechnological applications of retrons. (**A**) In retron-based bacterial gene editing, the msd is homologous to a segment of the target gene (pink). The desired mutation is also introduced into the msd (turquoise). msDNA (thin dashed/solid line) is produced and edits the targeted sequence via an undetermined mechanism. (**B**) Schematic of retron-mediated genomic recording via Synthetic Cellular Recorders Integrating Biological Events, e.g., ‘SCRIBE’ (13). A small-molecule inducible retron is designed to turn on or off a selectable marker. Switching of the selectable marker occurs only in the presence of the small molecule inducer (orange hexagon). The desired edit is produced in proportion to the duration and concentration of inducer, enabling ‘tape recording’ of the inducer's presence. (**C**) Retron-mediated mutagenesis and evolution (15). In this application, a retron homologous to the desired target gene is expressed under an error-prone T7 RNA polymerase (green). The resulting msDNAs have random mutations (stars) that are subsequently introduced into their target gene as in (A). Alternatively, these mutation-containing msDNAs can edit the parent retron sequence, enabling continuous accumulation of mutations and evolution. (**D**) Schematic for Cas9 Retron precISe Parallel Editing via homologY, e.g. ‘CRISPEY’ (14). A retron msr/msd sequence containing homology and a desired edit to a targeted gene is expressed as a fusion to a sgRNA (light blue) targeting the gene of interest. The retron RT (purple) and Cas9 (green) are expressed *in trans*. Cas9 generates a DNA break and the RT-generated DNA is used as a template by the host's DNA repair machinery. Genes are not drawn to scale.

More recently, a landmark study used retrons as bacterial genome-editing tools (13). In this work, small molecule-inducible retrons acted as a population-level genomic analog ‘tape-recorder’ of the small molecules’ presence in *E. coli* (Figure 4A, B). Termed Synthetic Cellular Recorders Integrating Biological Events (‘SCRIBE’), this approach used engineered variants of Retron-Eco1 (Ec86) with msds programmed to correct two premature stop codons in reporter genes, ultimately editing a total of 5–6 contiguous bases. Co-expressing the retron msr-msd with the bacteriophage lambda red protein Beta (a single-stranded DNA-binding protein commonly employed in recombineering (5)) increased editing frequency of the retron transcript a thousand-fold (from ∼10^−7^/cell to ∼10^−4^/cell) after overnight expression. This improved genome editing efficiency was appropriate for detection of modified cells within a heterogeneous population. SCRIBE could (i) quantitatively record inducers’ concentration and duration by measuring the proportion of cells containing retron-introduced edits; (ii) record exposure to complex combinations of inducer molecules and (iii) record cellular exposure to light via the use of a light-responsive promoter.

Retron-based editing in bacteria has been further adapted to support the possibility of continuous evolution of target loci (15) (Figure 4C). Knocking out MutS, previously shown to bind retron msDNA, further increased editing efficiency 21-fold compared to wild-type cells (70). Removing exonuclease ExoX, which degrades double and single-stranded DNAs from the 3′ to 5′ end, increased editing efficiency 5-fold (82). An *E. coli* that harbored the double *ΔmutSΔexoX* deletion increased retron-based editing efficiency 78-fold to ∼0.05/cell after overnight expression. Measurements with a similar kanamycin resistance-correcting model system to that employed in the bacterial tape recorder indicated that retrons could simultaneously edit up to 13 bases spanning a 31-base region, and insert and delete three-base regions. Expressing the retron in this optimized system under an error-prone RNA polymerase led to the introduction of unprogrammed, random mutations in the targeted gene, setting the stage for continuous evolution schemes. Overall, these modifications increased the mutation rate of the targeted region to ∼10^−6^ base^−1^ generation^−1^, ∼200-fold above background.

### Heterologous expression of retrons

Biotechnological retron applications were bolstered by early studies that retrons can produce satellite DNAs in eukaryotic hosts. For example, Retron-Eco2 (Ec67) expressed under the control of a Gal10 promoter produced msDNA in *S. cerevisiae* (83). Robust msDNA production required the concurrent expression of the RT and the msr-msd. The resulting msDNA was the same length as seen in the native *E. coli* host. In yeast, moving the RT to the 5′ side of the msr-msd region increased msDNA production sevenfold over that produced in the native orientation where the RT is to the 3′ side of the msr-msd region (83). Retron-Eco2 (Ec67) could also produce msDNA in mouse embryonic fibroblasts by expressing this 5′ RT-containing variant with T7 RNA polymerase (84). However, msDNA copy number was ∼25 copies per cell, ∼25-fold lower than seen in the native bacterial host. Nonetheless, these early studies established that retrons can be expressed in orthogonal hosts and that the host's RNAse H activity was sufficient to process the reverse-transcribed msd to generate msDNA.

More recently, retrons have been used for aiding homology-directed repair of Cas9-targeted breaks in yeast in a platform termed Cas9 Retron precISe Parallel Editing via homologY (‘CRISPEY’) (14) (Figure 4D). The msr-msd of Retron-Eco1 (Ec86) was appended to a Cas9 single guide RNA (sgRNA) that targeted a genomic region of interest. Co-expression of this chimeric sgRNA-retron RNA, RT-Eco1 (Ec86) and Cas9 led to the co-localization of two functional units: (i) a Cas9 nuclease that generated a defined DNA break in the vicinity of the edit site; (ii) a DNA template derived from the msd segment of the sgRNA-retron RNA fusion. After Cas9 and RT induction, the reverse-transcribed msd could carry out homology-directed DNA break repair at high efficiency. Single nucleotide polymorphisms were incorporated in >96% of all clones and a 765 base pair insertion was observed in 87% of edited yeast cells. Ultimately, this technique was employed for the high-efficiency profiling of the effect of ∼16,000 single-nucleotide polymorphisms in yeast, enabling identification of 572 variants that impacted fitness during growth on glucose. This approach can now potentially be applied to the combinatorial diversification of targeted regions, similar to combinatorial recombineering-based platforms such as MAGE (8), YOGE (9) or iCREATE (10).

## CONCLUSIONS AND OUTLOOK

A recent renaissance in retron research is fuelled by their broad phylogenetic distribution, intriguing potential linkages to pathogenesis, and unique biotechnological capabilities. Even with their newfound popularity, retron function, mechanism of action, and evolutionary origins remain obscure. One critical gap is the lack of a retron RT structure, either alone or as part of a priming complex. Thus, we can only conjecture about the unique priming mechanism and the functions of accessory domains in retron RTs. The functions of other retron-associated ORFs are also unexplored. As with many natural biomolecules that have proven indispensable to modern biotechnologies, an improved understanding of natural retron function and biology should greatly accelerate biotechnology applications.

Retrons are unique in their ability to produce satellite DNAs with arbitrary sequences *in vivo*. This is a feature that suddenly thrusts retrons to the fore of genome editing techniques, as they can be harnessed to enable genome editing without the need to introduce external oligonucleotides via electroporation or transfection. On the horizon are opportunities to harness retrons as dynamic, self-diversifying templates for editing targeted genes, thereby enabling targeted continuous evolution. Further optimizing retron expression and function in eukaryotic cells and organisms will likely enable diverse applications, such as the ability to mark cells during embryogenesis and carry out lineage analyses via continuous evolution of barcodes. In another vein, retron-produced DNA may also prove useful for generating highly programmable DNA nanostructures inside cells. Finally, retron-derived RTs may have unique applications on their own, just as Group II intron RTs have found applications for NGS library preparation.

## Supplementary Material

gkz865_Supplemental_File
