# Supplementary material for: Retrons and their applications in genome engineering
Source: Nucleic Acids Res. 2019 Oct 10;47(21):11007–19. doi: 10.1093/nar/gkz865 (PMC6868368; doi:10.1093/nar/gkz865)
Supplement: gkz865_Supplemental_File [file gkz865_supplemental_file.pdf]

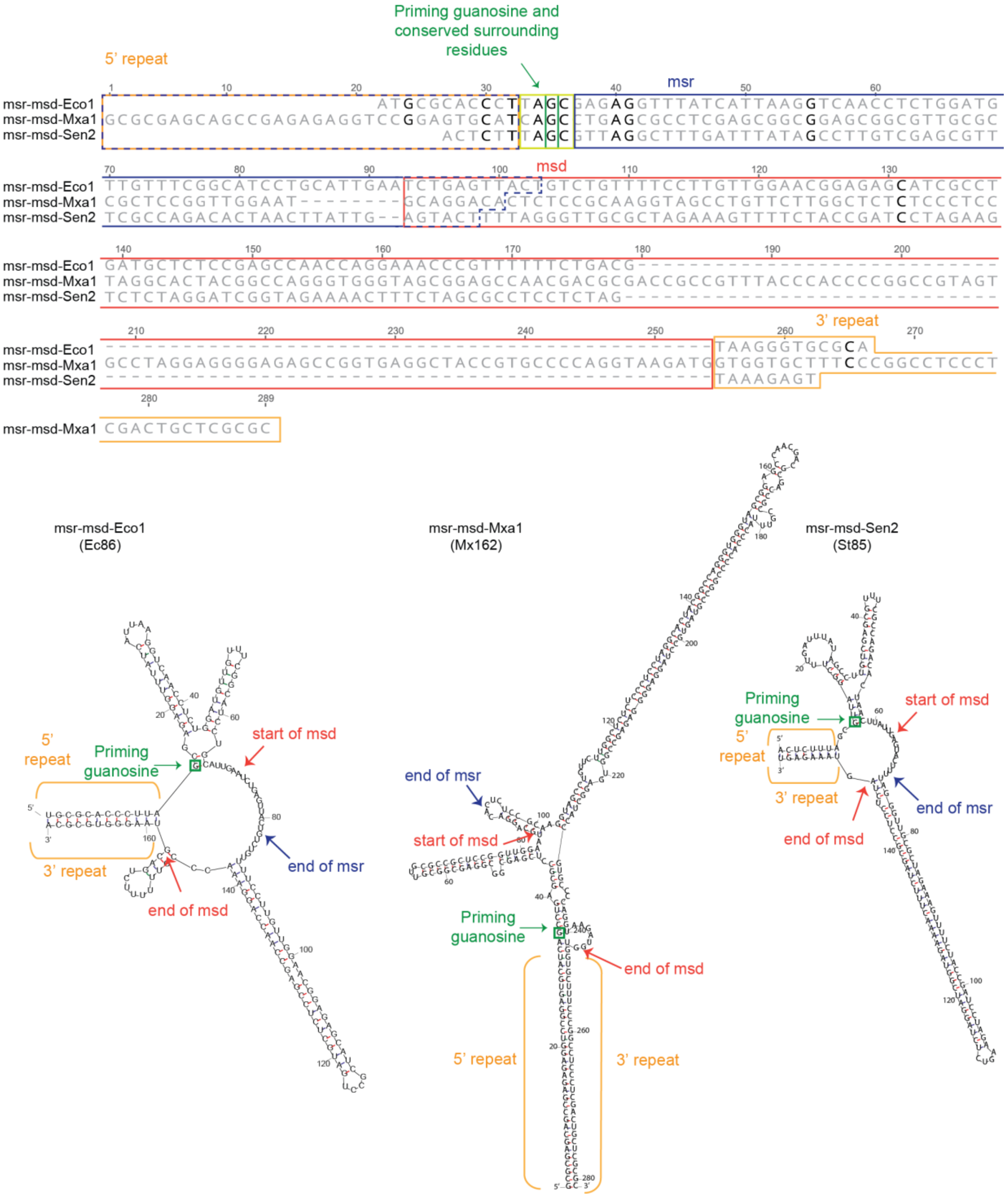

Figure S1. Msr-msd sequences and structures. Top: Alignment of representative msr-msd DNA sequences from Retron-Eco1 (Ec86), Retron-Mxa1 (Mx162), and Retron-Sen2 (St85). Bases that are conserved between all three sequences are in black; others are in grey. The 5' and 3' repeats, priming guanosines, conserved region surrounding the priming guanosines, msrs, and msds are boxed and labelled in orange, green, yellow-green, blue, and red, respectively. Bottom: Mfold-predicted most favorable msr-msd transcript structures (1).

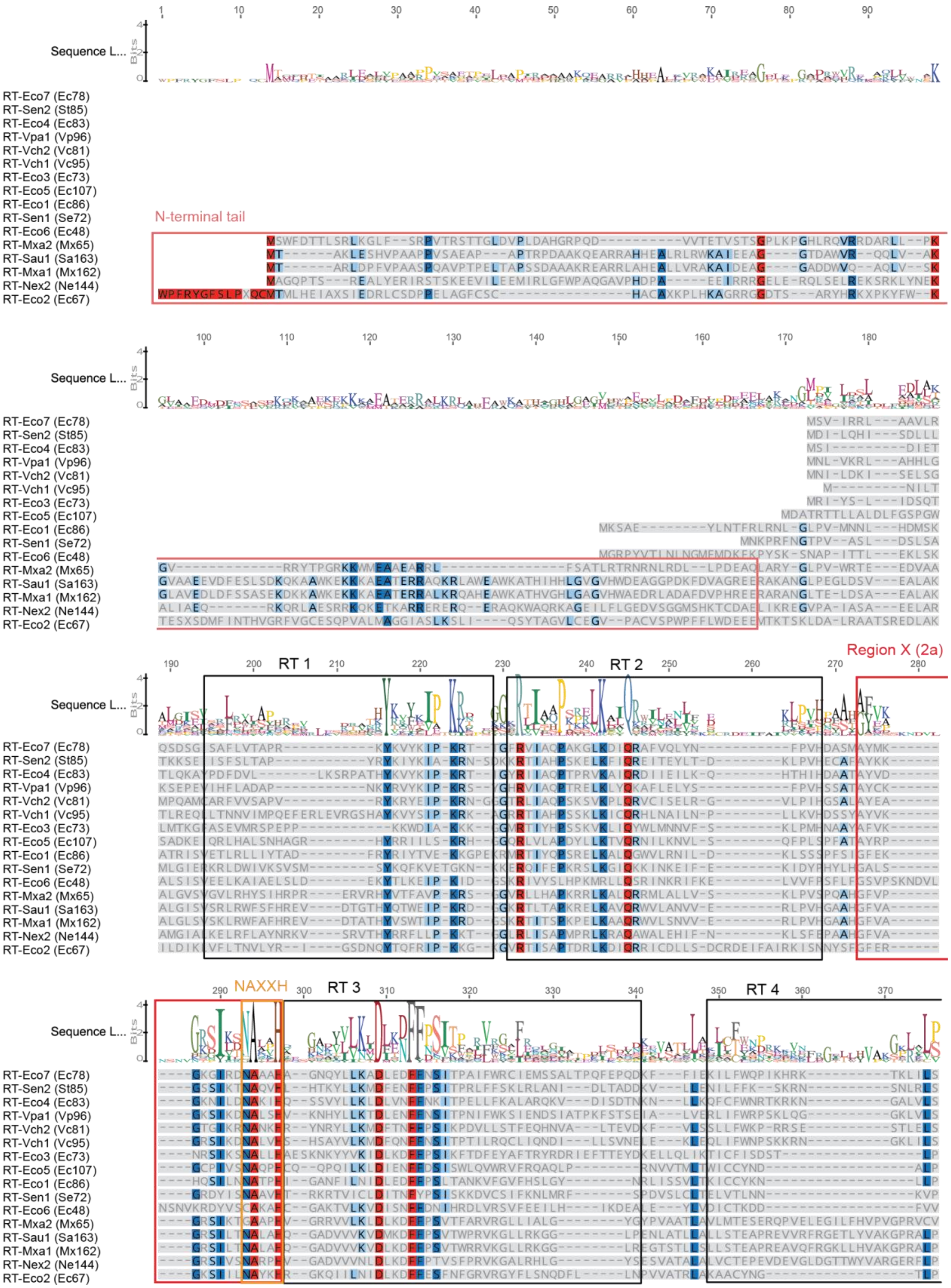
Sequence L...
Bits
N-terminal tail
RT 1
RT 2
Region X (2a)
NAXXH
RT 3
RT 4
RT-Eco7 (Ec78)
RT-Sen2 (St85)
RT-Eco4 (Ec83)
RT-Vpa1 (Vp96)
RT-Vch2 (Vc81)
RT-Vch1 (Vc95)
RT-Eco3 (Ec73)
RT-Eco5 (Ec107)
RT-Eco1 (Ec86)
RT-Sen1 (Se72)
RT-Eco6 (Ec48)
RT-Mxa2 (Mx65)
RT-Sau1 (Sa163)
RT-Mxa1 (Mx162)
RT-Nex2 (Ne144)
RT-Eco2 (Ec67)

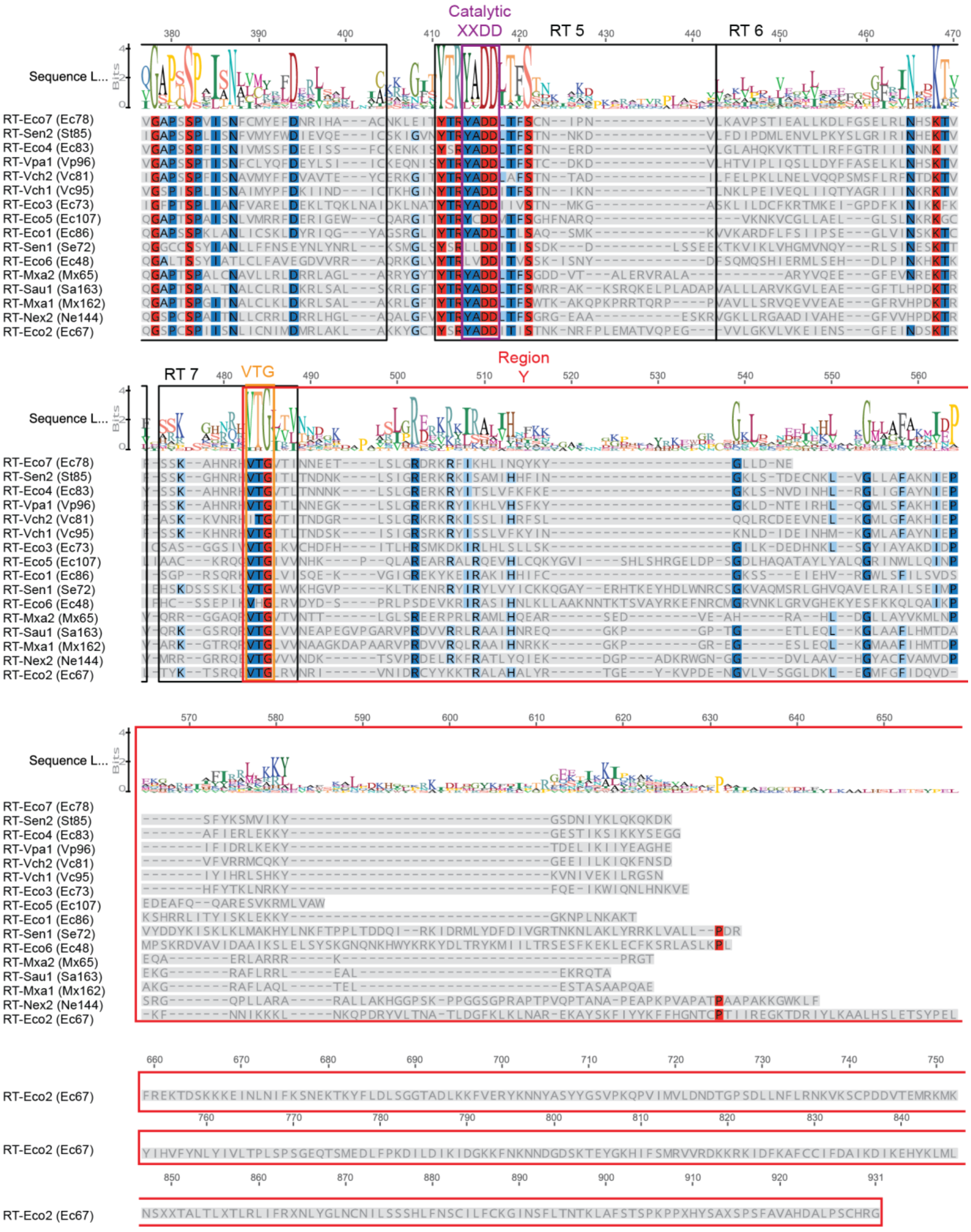

Figure S2. Alignment of all 16 experimentally validated retron RT sequences. Conserved RT regions are boxed in black, retron specific-regions in red, retron-specific motifs NAXXH and VTG in orange, and the catalytic XXDD tetrad in purple.

	Eco7 (Ec78)	Sen2 (St85)	Eco4 (Ec83)	Vpa1 (Vp96)	Vch2 (Vc81)	Vch1 (Vc95)	Eco3 (Ec73)	Eco5 (Ec107)	Eco1 (Ec86)	Sen1 (Se72)	Ec4 (Ec48)	Mxa2 (Mx65)	Sau1 (Se163)	Mxa1 (Mx162)	Nex2 (Ne144)	Eco2 (Ec67)
**Eco7 (Ec78)**		43.8	42.0	58.2	42.0	42.0	27.7	24.8	23.8	15.4	15.9	21.2	22.3	23.8	19.7	21.8
**Sen2 (St85)**	43.8		42.9	43.4	49.2	45.5	31.7	22.3	28.3	18.0	14.3	21.3	22.1	23.5	16.8	20.4
**Eco4 (Ec83)**	42.0	42.9		45.8	39.8	45.7	27.0	22.8	23.4	16.0	13.5	20.1	20.3	21.4	18.2	18.9
**Vpa1 (Vp96)**	58.2	43.4	45.8		41.2	43.3	26.6	23.6	24.5	16.9	13.7	21.8	20.9	22.8	16.9	17.8
**Vch2 (Vc81)**	42.0	49.2	39.8	41.2		39.9	28.4	22.2	22.0	16.1	14.8	21.0	21.5	24.0	16.7	18.6
**Vch1 (Vc95)**	42.0	45.5	45.7	43.3	39.9		27.2	19.7	23.1	16.1	13.9	20.7	20.7	21.4	16.5	19.5
**Eco3 (Ec73)**	27.7	31.7	27.0	26.6	28.4	27.2		21.3	22.8	14.5	15.1	21.6	22.3	22.0	20.6	18.7
**Eco5 (Ec107)**	24.8	22.3	22.8	23.6	22.2	19.7	21.3		24.0	22.0	19.6	24.4	25.7	23.0	24.5	22.2
**Eco1 (Ec86)**	23.8	28.3	23.4	24.5	22.0	23.1	22.8	24.0		20.4	18.1	24.7	24.8	26.2	25.4	25.8
**Sen1 (Se72)**	15.4	18.0	16.0	16.9	16.1	16.1	14.5	22.0	20.4		25.1	17.5	17.7	17.5	19.2	19.6
**Ec6 (Ec48)**	15.9	14.3	13.5	13.7	14.8	13.9	15.1	19.6	18.1	25.1		15.7	16.9	18.4	16.2	16.6
**Mxa2 (Mx65)**	21.2	21.3	20.1	21.8	21.0	20.7	21.6	24.4	24.7	17.5	15.7		34.5	33.3	31.4	19.9
**Sau1 (Se163)**	22.3	22.1	20.3	20.9	21.5	20.7	22.3	25.7	24.8	17.7	16.9	34.5		73.0	35.4	23.4
**Mxa1 (Mx162)**	23.8	23.5	21.4	22.8	24.0	21.4	22.0	23.0	26.2	17.5	18.4	33.3	73.0		34.5	23.3
**Nex2 (Ne144)**	19.7	16.8	18.2	16.9	16.7	16.5	20.6	24.5	25.4	19.2	16.2	31.4	35.4	34.5		21.7
**Eco2 (Ec67)**	21.8	20.4	18.9	17.8	18.6	19.5	18.7	22.2	25.8	19.6	16.6	19.9	23.4	23.3	21.7	

Table S1. Comparison of retron RT sequence identity. Identity was compared by aligning all RT sequences in NCBI's online Constrain-based Multiple Alignment Tool (COBALT) (2), with the following parameters: gap penalty opening: -11; gap penalty extension: -1; end gap opening: -5; end gap extension: -1; RPS BLAST to guide alignment on; constrain E-value 0.003; find conserved columns and recompute alignment on; use query clustering parameters on; word size 4; max cluster distance 0.8; and alphabet regular.

REFERENCES

1. Zuker, M. (2003) Mfold web server for nucleic acid folding and hybridization prediction. *Nucleic Acids Research*, **31**, 3406-3415.
2. Papadopoulos, J.S. and Agarwala, R. (2007) COBALT: constraint-based alignment tool for multiple protein sequences. *Bioinformatics*, **23**, 1073-1079.
